# Pulmonary Embolism Incidence and Fatality Trends in Chinese Hospitals from 1997 to 2008: A Multicenter Registration Study

**DOI:** 10.1371/journal.pone.0026861

**Published:** 2011-11-01

**Authors:** Yuanhua Yang, Lirong Liang, Zhenguo Zhai, Hangyong He, Wanmu Xie, Xiaoxia Peng, Chen Wang, on behalf of investigators for the National Cooperative Project for the Prevention and Treatment of PTE-DVT

**Affiliations:** 1 Beijing Key Laboratory of Respiratory and Pulmonary Circulation Disorders, Beijing Chao-yang Hospital, Capital Medical University, Beijing, China; 2 Beijing Institute of Respiratory Medicine, Beijing Chao-yang Hospital, Capital Medical University, Beijing, China; 3 Department of Epidemiology and Biostatistics, School of Public Health and Family Medicine, Capital Medical University, Beijing, China; 4 Beijing Municipal Key Lab of Clinical Epidemiology, Beijing, China; 5 Beijing Hospital of the Ministry of Health, Beijing, China; University of Hong Kong, Hong Kong

## Abstract

**Background:**

There has not been sufficient evidence to support the Asians being less susceptible to pulmonary embolism (PE) than other ethnicities, because the prevalence of PE/deep venous thrombosis (DVT) in different racial and ethnic groups has not been carefully studied until recently except in Caucasians. To test the hypothesis that the Chinese population has a lower risk for PE, this study comprehensively assessed the hospital-based incidence and case fatality rates for PE during the 1997–2008 in China.

**Methods:**

A registration study of patients with suspected PE syndromes admitted to 60 level-3 hospitals involved in the National Cooperative Project for the Prevention and Treatment of Venous Thromboembolism (NCPPT) was conducted from January 1997 to December 2008. The only exclusion criterion was an age of less than 18 years. Helical computed tomography scan, ventilation-perfusion lung scintigraphy or pulmonary angiography was carried out before or after hospitalization. All images were reviewed and evaluated independently by two specialists.

**Results:**

A total of 18,206 patients were confirmed with PE from 16,972,182 hospital admissions. The annual incidence was 0.1% (95% CI: 0.1% to 0.2%). The overall incidence of PE in male patients (0.2%, 95% CI: 0.1% to 0.3%) was higher than that in female patients (0.1% and 95% CI: 0.0% to 0.1%). An increasing incidence gradient for PE was noticed from Southern to Northern China. In addition, the case fatality rate was apparently decreasing: 25.1% (95% CI: 16.2% to 36.9%) in 1997 to 8.7% (95% CI: 3.5% to 15.8%) in 2008.

**Conclusions:**

Our findings suggest the relatively stable PE incidence and decreasing fatality trends in Chinese hospitals may be partially attributable to the implementation of the NCCPT and suggest the government should reevaluate the severity of PE so that health resources for the prevention, diagnosis and treatment of PE could be used to their fullest.

## Introduction

In the United States, the incidence of pulmonary embolisms (PE) among hospitalized patients from 1979 to 1999 was a stable 0.4% of admissions [1]. PE comprised more than 30.0% of all venous thromboembolism (VTE) [2], [3], the third most common cardiovascular disease in the United States after acute ischemic cardiovascular syndromes and stroke [4]. During the past several decades, considerable progress has been made in the prevention and treatment of PE, resulting in a substantial decline in PE-specific mortality from 45 to 33 deaths per million persons [5].

In Asia, studies showed that the directly standardized incidence (number of cases within a population comprised of a single racial group) of all VTE events was strikingly lower among Asians/Pacific Islanders (29/100,000/yr) than among Caucasians (103/100,000/yr) [6], [7]. Compared to Caucasians, the incidence of VTE in Asians/Pacific Islanders was approximately 3-fold lower for secondary VTE events and 5-fold lower for idiopathic VTE events, and a similar incidence of VTE among Asians has been reported in Hong Kong [8], [9] and Japan [10].

Since the presence of factor V Leiden or the prothrombin gene mutation (G20210A) is known to be associated with a 3 to 4-fold higher risk for developing VTE [11], [12], it is logical to assume that the prevalence of VTE would be lower in Asian populations than in Caucasian populations, because the prevalence of these two genetic traits is much lower in Asian populations than Caucasian populations [13]. However, some studies have suggested that the incidence of asymptomatic VTE may not differ substantially between Asians/Pacific Islanders and Caucasians [14], [15]. In fact, the conjecture that Asians' susceptibility to PE is less than other ethnic groups has not been sufficiently supported because of a lack of careful studies into the prevalence of VTE/PE among ethnic groups other than Caucasians.

In China, the National Cooperative Project for the Prevention and Treatment of Venous Thromboembolism (NCPPT) [16] was initiated in 2001 to improve the awareness among clinicians and to standardize the management practice for the diagnosis and treatment of VTE. A total of 60 level-3 hospitals (major tertiary referral centers in provincial and major urban hospitals) were recruited, covering 22 provinces and 2 autonomous cities (Beijing and Shanghai). A multicenter registry of patients admitted to hospitals with suspected VTE syndromes was initiated among the 60 hospitals, allowing the NCPPT to gather valuable information about the epidemiology of PE. In this first comprehensive assessment of the incidence and case fatality rate of PE in Chinese hospitals from 1997 to 2008, the hypothesis that the Chinese population has a lower risk for PE was confirmed^17^. This information will help to maximize the application of both public health and clinical resources to the prevention, diagnosis and treatment of PE.

## Methods

### Registry Design and Data collection

The NCPPT project in China involved a multicenter registry of patients admitted to hospitals with suspected VTE syndromes as well as a multicenter randomized and controlled trial to evaluate the efficacy and safety of PE anticoagulation and prophylaxis among Chinese patients. The institutional review boards at Beijing Chao-Yang Hospital, Capital Medical University approved the protocol. Written, informed consent was obtained for all prospective data collected for the purposes of this study prior to the onset of the study. Consent was not necessary for retrospective data used in this study because the data were analyzed anonymously.

Between January of 1997 and December of 2008, consecutive patients admitted to the inpatient ward with a diagnosis of suspected PE were registered from 60 hospitals (see the Appendix), covering 22 provinces and 2 autonomous cities (Beijing and Shanghai). The only exclusion criterion was an age of less than 18 years.

All participating hospitals were major provincial-level tertiary referral centers or major urban hospitals with facilities for conducting helical computed tomography scan, ventilation-perfusion lung scintigraphy, pulmonary angiography and echocardiogram visualization of thrombus. A registry coordinator controlled the quality of data collection (e.g. internal validity and coherence) at each participating center and recorded the data from each patient on a case report form. Specialist staff employees skilled in cardiovascular and pulmonary diseases, helical computed tomography scanning, pulmonary angiography and ultrasound were involved in the diagnosis and treatment of the PE patients after standardized training according to our standard protocol. Coordinators and specialists ensured that all consecutive patients with conformed PE were included in the registry.

The data from January of 1997 to December of 2000 was collected retrospectively by a review of the medical records. For this study, the following data were collected prospectively from 2001: demographic data, types and results of diagnosis methods and prognosis. Raw data were checked then forwarded to the Study Coordinating Centre in Beijing Chao-yang Hospital where data were input into a duplicate computerized database and then checked again.

### Study Outcome

All patients were identified with a discharge diagnosis of PE (first-episode or recurrent PE) based on the St. Anthony's International Classification of Disease, Ninth Revision (ICD-9) diagnostic codes, including 415.1 (pulmonary embolism and infarction), 415.11 (iatrogenic pulmonary embolism and infarction), 415.19 (pulmonary embolism other), 634.6, 635.6, 636.6, 637.6, and 638.6 (pulmonary embolism with abortion), 639.6 (pulmonary embolism with ectopic pregnancy), and 673.2 (pulmonary embolism with pregnancy, childbirth or the puerperium).

Helical computed tomography scan, ventilation-perfusion lung scintigraphy or pulmonary angiography was carried out before or after hospitalization. All images were reviewed and evaluated independently by two specialists using methods from previous studies [17], [18], [19]. Death was considered to be attributable to PE if the diagnosis was documented at autopsy or if the patient died shortly after an objective confirmation of symptomatic PE along with an absence of an alternative diagnosis. An independent outcome committee composed of four specialists in pulmonary circulation (Wang Chen, Yang Yuanhua, Chen Xiansheng and Lu Weixuan) reviewed and adjudicated all study outcomes.

### Analysis

Data management and statistical analysis were conducted by SAS software version 9.1.3 (SAS Institute Inc, Cary, NC, USA, 2006). Incidences (number of diagnoses/100 hospitalizations) of PE in hospitalized adults (aged 18 years and above) were calculated as the number of patients with the diagnosis divided by the number of hospital discharges from 1997 to 2008 multiplied by 100 annually. The annual case fatality rate for PE was calculated as the number of deaths from PE per 100 hospitalized PE patients. Both the incidence and the case fatality rate were stratified into 10-year age groups, gender and geographical region (Northern China and Southern China according to the Huai River-Qinling Mountain line). Within these strata, the rates were estimated with 95% confidence intervals (CI) using the accurate calculation of CI for Poisson distribution. A chi-square test was used to compare the various frequencies with α<0.05. Poisson regression analysis was used to evaluate the relative risk (RR) for the incidence and fatality of PE when the age, gender and regional differences were compared.

## Results

Among the 60 hospitals, 21 hospitals were located in the largest cities in China, including Beijing and Shanghai. The remaining hospitals were located in provincial capitals or other major cities. The participating hospitals were widely distributed across the country, covering Northeastern China (5 hospitals), Northern China (21 hospitals), Central-Southern China (14 hospitals), Eastern China (12 hospitals), Southwestern China (4 hospitals) and Northwestern China (4 hospitals). From January of 1997 to December of 2008, hospitalization data were collected from 18,206 PE diagnosed patients from a total of 16,972,182 discharged patients throughout the participating hospitals. A total of 13,137 PE patients from 8,289,509 hospital admissions in 36 hospitals located in Northern China and 5,069 patients from 8,682,673 hospital admissions in 24 hospitals located in Southern China were detected. Among these 18,206 patients with PE, patients aged 50 and above were more frequent (70.3%). In addition, male patients (10,425) accounted for 57.3% of all patients.

The age-specific incidence of PE increased with age, especially for aged male patients **(** **Table 1**). The incidence of PE among patients 70 years old and above was 0.4% (95% CI: 0.3% to 0.6%). The overall incidence of PE in male patients (0.2% and 95% CI: 0.1% to 0.3%) was higher than that in female patients (0.1% and 95% CI: 0.0% to 0.1%). However, the discrepancy between genders was not statistically significant, because the 95% CI overlapped.

**Table 1:** The incidence of PE in hospitalized patients by age and gender in China (%) during 12 years (from 1997 to 2008).

Age	Total	Men	Female
< = 30	0.07(0.02–0.13)	0.12(0.06–0.21)	0.05(0.02–0.11)
31–40	0.06(0.02–0.12)	0.14(0.08–0.23)	0.03(0.01–0.09)
41–50	0.13(0.07–0.22)	0.16(0.09–0.25)	0.10(0.05–0.18)
51–60	0.12(0.06–0.21)	0.14(0.08–0.23)	0.10(0.05–0.18)
61–70	0.14(0.08–0.23)	0.19(0.11–0.29)	0.12(0.06–0.21)
71+	0.10(0.05–0.18)	0.44(0.32–0.59)	0.05(0.02–0.11)
Total	0.11(0.05–0.19)	0.18(0.10–0.28)	0.07(0.02–0.13)

**Figure 1:**
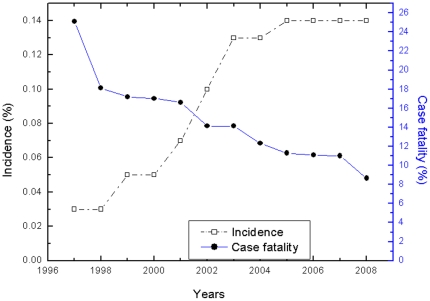
Incidence and case fatality rates for PE in hospitalized adults from 1997 to 2008.

The annual incidence increased sharply from 0.0% (95% CI: 0.0% to 0.1%) in 1997 to 0.1% (95% CI: 0.1% to 0.2%) in 2003, then remained at 0.1% (95% CI: 0.1% to 0.2%) (**Figure 1**). Conversely, the case fatality rate decreased from 25.1% (95% CI: 16.2% to 36. 9%) in 1997 to 8.6% (95% CI: 3.5% to 15.8%) in 2008 (**Figure 1**).

**Figure 2:**
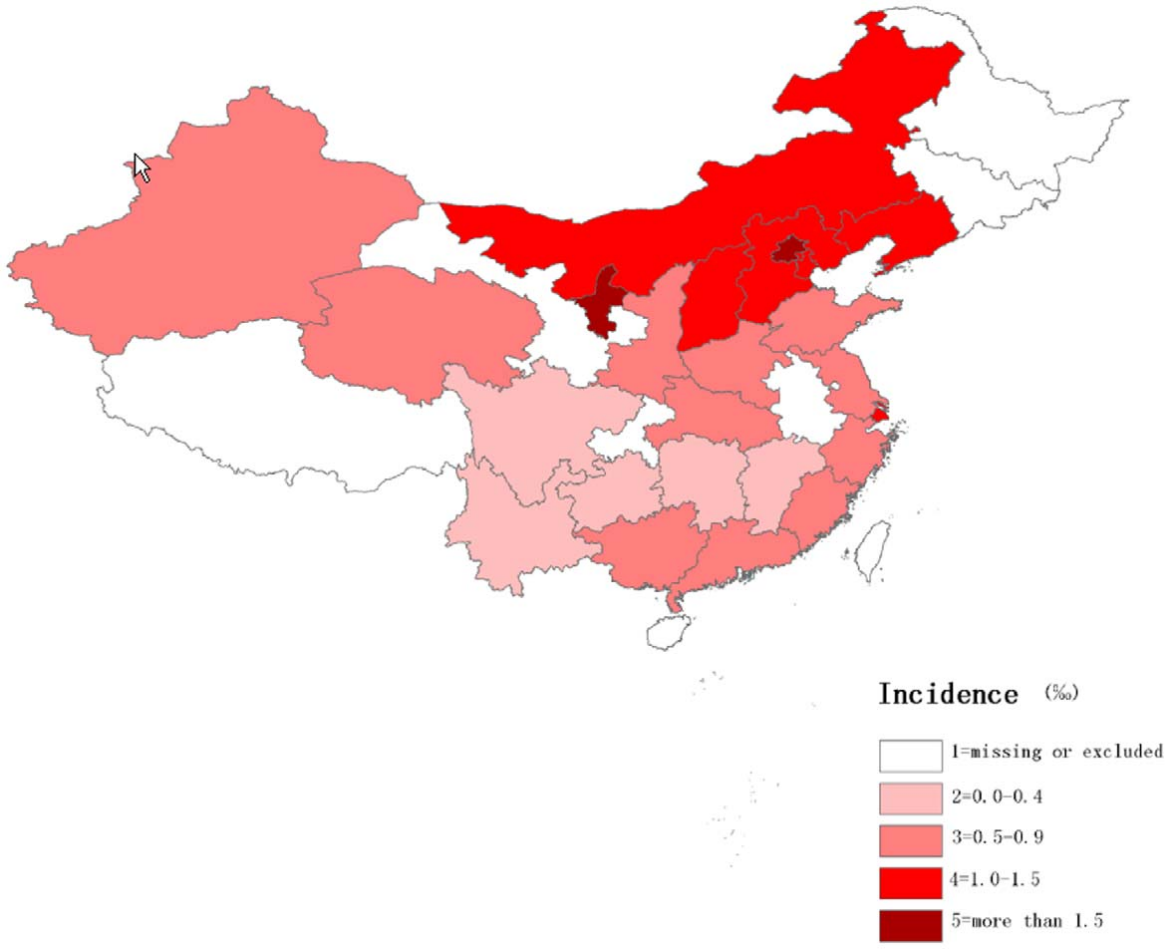
The regional distribution of incidence for PE in China.

**Figure 3:**
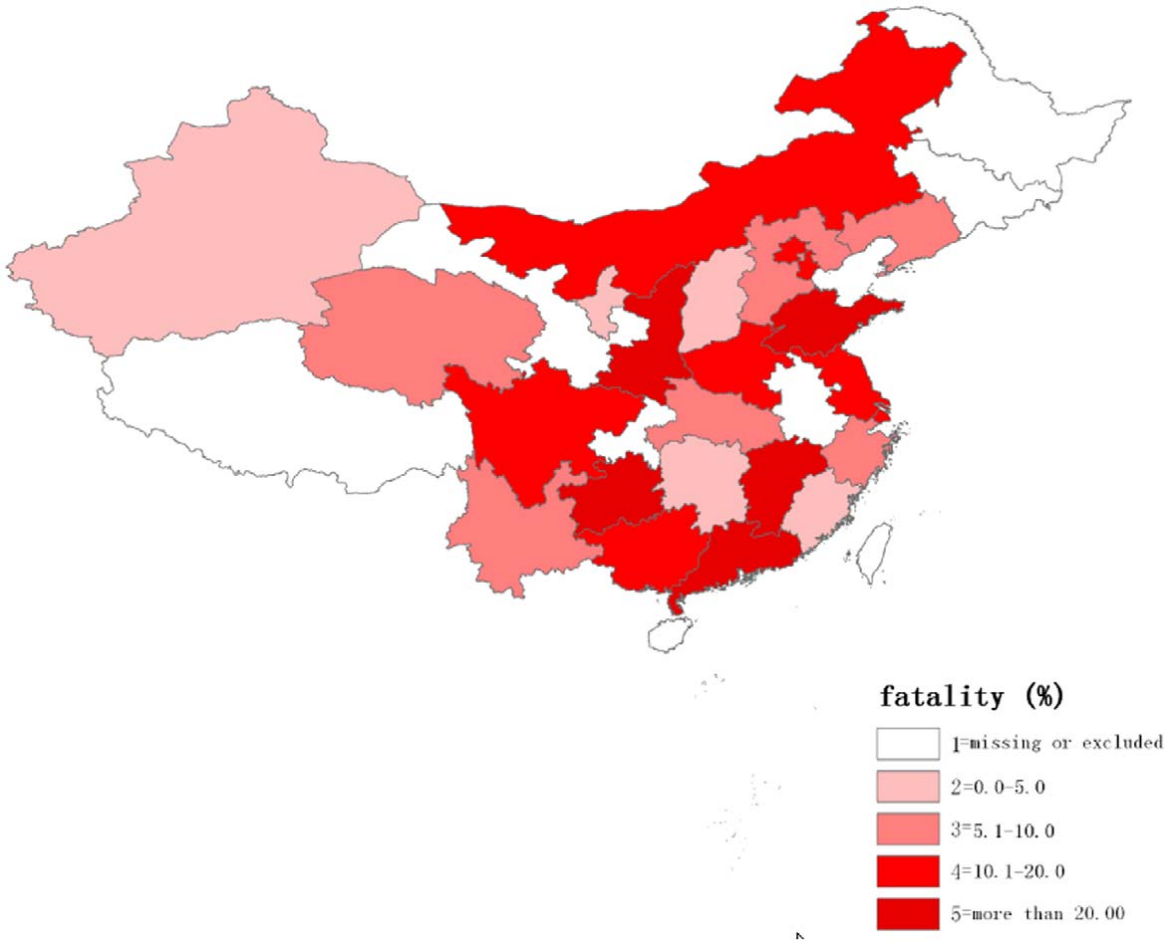
The regional distribution of case fatality rates for PE in China.

**Figure 4:**
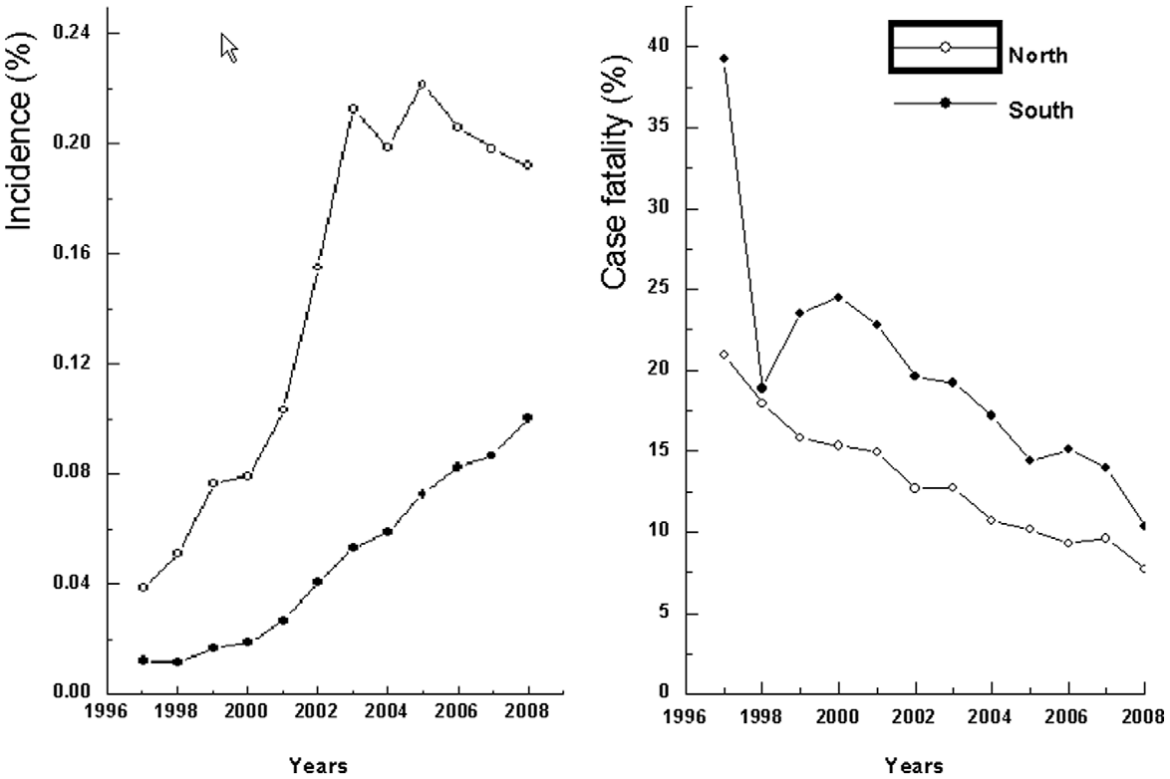
The discrepancy between the North and the South in incidence and case fatality rates for PE in China from 1997 to 2008.

In addition, the region-specific incidences and case fatalities were mapped in **Figure 2** and **Figure 3** respectively. The incidence of PE increased from Southern to Northern China, whereas the case fatality rate decreased. A further analysis of the discrepancy between the North and the South showed that the incidence of PE in hospitalized patients in Northern China (0.2%) over the 12 year period were higher than in Southern China (0.1%) (**Figure 4**). Poisson regression also provided evidence for a difference in incidence of PE between Northern and Southern China, the value of relative risk (RR) being 2.7 (95% CI: 2.6 to 2.8). In contrast, the case fatality rate for PE was lower in the North (10.9%) than in the South (15.3%) with an RR value of 0.7 (95% CI: 0.6 to 0.8).

## Discussion

From this multicenter registration study on the incidence and case fatality rate of PE, we found that the annual incidence increased sharply from 0.0% (95% CI: 0.0% to 0.1%) in 1997 to 0.1% (95% CI: 0.1 to 0.2) in 2003, and remained at 0.1% (95% CI, 0.1% to 0.2%) through 2008, suggesting that the incidence of PE was lower in Chinese hospitals than in USA hospitals (0.4%) [1], [20]. In addition, an increase in PE from the South to the North was shown in the present study and the incidence of PE was higher in male patients than in female patients.

Our study revealed that 69.2% of PE patients were from 41 to 70 years old and that in this age range the incidence for PE was about 0.1%; lower than findings reported by Stein [21] (0.2% for patients aged 40 to 59; 0.3% for patients aged 60 to 69). Previous studies have shown that the incidence of first-time VTE rises exponentially with age, increasing dramatically after 60 years of age [22], [23]. The incidence of PE in Chinese men increased exponentially after 70 years of age (0.4%), dramatically higher than other age groups (0.1%–0.2%). Conversely, the incidence of PE in female patients declined sharply from 0.10 (40–69 age group) to 0.05% after 70 years of age. The effect of gender on the incidence of PE observed in our study was not consistent with other studies. Stein and Anderson reported a similar incidence in both sexes [1], [24]. But Silverstein noted a higher age-adjusted incidence among males than females (0.13% vs 0.11%, respectively) [22]. Kniffin also reported that among patients aged 65 years and above women had a lower risk of PE and the value of RR was 0.9 (95% CI, 0.8 to 0.9) [25]. The strikingly lower incidence of PE among elder Chinese women may be because of the lower hospitalization rates for elder Chinese women (6.5% vs 8.8% in 2003; 12.9% vs 14.8% in 2008) than elder Chinese men [26], less prevalent use of oral contraceptives and postmenopausal hormone replacement.

In addition, the present study has shown an increase in the annual incidence of PE from 1997 to 2004. The potential explanations are: (1) an increased rate of detection of PE because of the implementation of NCPPT; (2) the increased availability and use of diagnostic technologies such as ventilation-perfusion lung scintigraphy and computed tomographic pulmonary angiography (CTPA). The incidence of PE was lower in hospital patients in China than in the USA (0.3% to 0.4% in hospitalized patients in large urban hospitals, major university hospitals and community/teaching general hospitals) [27]–[29]. These differences may be partly because of a difference in risk-level factors for PE and a difference in the accuracy of diagnoses. The previously reported findings among Hong Kong Chinese (0.04%, about one-tenth of western communities) [8], [9] suggested the possibility of an underestimation of risk for PE among the Chinese population. Therefore, our findings demonstrate that the difference in the incidence of PE between Chinese and Western populations is smaller than that previously reported.

However, as the incidence of PE increased, the case fatality rate for PE declined annually. Stein et al. reported that the incidence of PE among hospitalized adults aged ≥20 years did not change during the 1979 to 1999 period [1]. In addition, he also found that the diagnosis rate decreased over that period [30]. Lilienfeld et al. reported that the population mortality for PE declined over the same period [31]. However, Stein et al. reported that the case fatality rate for PE increased from 1979 to 1989, and then decreased from 1990 to 1998. The authors suggested that the declining population mortality rate for PE from 1979 to 1989 could be because of a decreased incidence of PE from improved prophylaxis; whereas the continuing decline in population mortality rate for PE through 1998 reflected a decreased case fatality rate from earlier and better management [3]. The drop in PE case fatality rate found in this study may be because of economic development, social progress and improvements in medical treatment and services. More importantly, however, the implementation of NCPPT [16] may have improved awareness as well as the quality of detection and management of patients with VTE.

An increase in the incidence of PE from the South to the North was discovered in our study, concomitant with a slight decline in the case fatality rate. Although this regional difference in the incidence of PE has not been discussed in previous studies, a South to North difference in coronary heart disease and stroke in China has been reported [32]. This difference was attributed to regional differences in cardiovascular risk factors, including hypertension, dyslipidemia, obesity and diabetes [33]–[35]. It has been shown that these factors are also associated with an increased risk for PE [36]. However, differences in levels of detection and treatment for PE between the North and the South may contribute to the reported regional difference in the case fatality rate for PE.

The present study has several limitations. Our findings were based entirely on discharge data rather than on the general population, so the national epidemiological trends for PE could not be evaluated more precisely. Second, the protocol was not designed to track multiple admissions for individuals and thus failed to evaluate the incidence of first episode PE. Third, our discharge data did not contain the patients that had died outside of the hospital, at home or in a nursing facility, because it was assumed that only a small number of those suffering from PE were likely to seek medical care and thus be admitted to the hospital. Moreover, our data are exclusively derived from tertiary care hospitals (major tertiary referral centers in provincial hospitals and major cities) and do not involve any information from secondary care hospitals, in which the incidence of PE would be lower and the fatality would be higher as a result of poor diagnostic and treatment facilities for PE. Finally, we were unable to explore the effects from factors other than age, gender and region by multivariate Poisson regression analysis in this study because of a limited access to risk factor information for PE (socioeconomic status, obesity etc.).

Despite these limitations, our findings suggested a rising and then relatively stable incidence of PE among hospital patients in China (0.1%) and a declining annual case fatality rate, partly because of the implementation of NCCPT. In addition, an increase in the incidence of PE from the South to the North was shown in this study and the incidence of PE was higher in male patients than in female patients. The evidence suggests that the substantial decline in PE-specific mortality and the annual age-adjusted mortality from PE is a result of improved prevention and treatment for PE [5]. Therefore, this first comprehensive assessment on the incidence and case fatality of PE in hospital patients during the 1997–2008 period in China should encourage the government to reevaluate the burden of disease from PE so that both public health and clinical resources for the prevention, diagnosis and treatment of PE will be used to their fullest. Moreover, the number one priority in the future prevention and treatment of PE in China may be to reduce the regional differences between the North and the South.
